# Changes in Individual Weight Status Based on Body Mass Index and Waist Circumference in Hong Kong Chinese

**DOI:** 10.1371/journal.pone.0119827

**Published:** 2015-03-16

**Authors:** Lai Ming Ho, Man Ping Wang, Sai Yin Ho, Tai Hing Lam

**Affiliations:** 1 School of Public Health, University of Hong Kong, Hong Kong, China; 2 School of Nursing, University of Hong Kong, Hong Kong, China; School of Public Health, Zhejiang University, CHINA

## Abstract

**Background:**

Weight change predicted diseases and mortality. We investigate 3-year changes in individual body mass index (BMI) and waist circumference in Hong Kong Chinese adults.

**Methods:**

In the Population Health Survey, 7084 adults in 2003 (baseline) were followed up in 2006. Longitudinal anthropometric data were available in 2941 (41.5%) for BMI and 2956 for waist circumference. Weight status and central obesity were based on objectively measured BMI and waist circumference using Asian standards.

**Results:**

Mean BMI (SD) increased from 22.8 (3.62) to 23.1 (3.95) (p<0.001) with 1.3 percentage point increase in prevalence of overweight and obesity (from 44.3% to 45.6%). One in 5 (22.0%) normal or underweight baseline respondents became overweight or obese and a similar proportion (24.8%) of overweight and obese respondents became normal or underweight. Prevalence of central obesity increased from 28.3% to 32.4% (p<0.001) with a non-significantly greater increase in women (30.0% to 38.1%) than men (23.0% to 26.1%) (p=0.63). A higher proportion of centrally obese respondents returned to normal (29.4%) than normal respondents developing central obesity (17.4%).

**Conclusions:**

This is one of the few studies in Chinese, which found dynamic longitudinal changes (increase/stable/decrease) in individual weight status and waist circumference. Future studies with better follow-up and investigating the causes of such changes are warranted.

## Introduction

Obesity is increasingly prevalent in developed and developing countries. In mainland China, the prevalence of general (body mass index, BMI, ≥25) and central obesity increased from 3.4% and 18.6% in 1993 to 26.4% and 37.4% in 2009, respectively [1]. Assessments on time trends of obesity are based on repeated cross-sectional surveys but comparability of data from surveys in different years using different samples can be problematic due to variation in methods and sampling errors. Moreover, apparent stable or changing prevalence rates could be the result of dynamic changes of individual weight status between obese and non-obese, which can only be revealed using longitudinal data.

Although Hong Kong is the most westernised city in China, the government’s Behavioural Risk Factor Surveillance showed a stable prevalence of self-reported general obesity (around 20%) in the past decade (2004–2012) [2]. Compared with general obesity, less is known about central obesity in Chinese people. A cross-sectional study in Hong Kong reported that the prevalence of central obesity increased in men (from 23.0% in 2001 to 26.9% in 2005) and remained stable in women [3]. Central obesity among Chinese adults was associated with risk factors of cardiovascular diseases, hypertension and diabetes [4–6]. Changes in individual weight status and waist circumference were not examined in the studies above, although in the West, these are increasingly found to better predict diseases and mortality than static baseline measures [7,8].

Among the few longitudinal studies examining individual weight changes, most were based on Western populations and focused on weight gain among normal weight subjects at baseline [9–12]. Although modest weight loss (≤10%) was found to reduce risk of cardiovascular diseases, diabetes and mortality [13,14], less is known about the population pattern on individual weight loss. Weight fluctuation or cycling, meaning repeated weight loss and regain, was associated with some negative health outcomes [15], but the effect on mortality is unclear. Studies on trajectory of weight changes have identified four types of trajectory: normal, overweight, late adulthood obesity and early adulthood obesity, and found most people (98% men and 92% women) had upward sloping trajectories [16,17]. Our review of the literature found no report focusing on individual weight changes in Chinese. In the present study, we describe the individual changes in body weight and waist circumference in Chinese adults in Hong Kong.

## Methods

### Ethical statement

Ethical approval was granted by Institutional Review Board (IRB) of the University of Hong Kong/Hospital Authority Hong Kong West Cluster. Written informed consents were obtained from the adult respondents or parent of respondents aged 18 or below, and the procedure was approved by the IRB.

### Baseline survey

The Hong Kong Population Health Survey (PHS) in 2003/2004 aimed to collect population representative data to support health policy. Respondents were selected using probability sampling based on the frame of quarters maintained by the Hong Kong Government Census and Statistics Department. Face-to-face interviews were conducted in 3035 households with 7084 land-based non-institutionalised Chinese-speaking respondents aged 15+. We included respondents aged 15–17 as to strengthen the representativeness of our findings to general population in Hong Kong, which usually covers this age period. The response rate was about 72% at household level and 44% at individual level. A structured questionnaire and anthropometric measures were used by trained interviewers to collect data on patterns of health status, health knowledge, attitudes and behaviours. Details of the survey methods and baseline main results have been published elsewhere [18].

### Follow-up survey

The 3-year follow-up survey of all baseline PHS respondents were done in 2006. The households were contacted by mail and telephone to obtain informed consent. Survey methods were the same as in 2003/4 to allow for comparison of time trend. Of 3035 households interviewed at baseline, 573 (18.9%) were invalid at follow-up. These included 489 moved households, 64 unoccupied quarters, 13 deceased respondents and 7 non-residential addresses. Of 2462 (81.1% of 3035) valid households, 1251 were fully enumerated and 266 were partially enumerated, representing 61.6% of the 2462 valid addresses. The refusal rate was 17.0% and non-contact rate was 21.4%. A total of 3131 subjects (44.2% of 7084) were successfully re-interviewed.

### Study measures

Body weight, height and waist circumference (WC) were measured at respondents’ homes by trained interviewers using valid, reliable and calibrated instruments, namely TANITA precision bathroom scale (Model HA-521), 150cm tailoring ruler, and 3.5m foldable metallic measuring tapes. Information about demographics and other characteristics was collected including sex, age, place of birth, education, income and marital status. In the present study, the main outcome variables were overweight, obesity and central obesity as defined by the World Health Organisation (WHO) Asian standard for BMI and WC [19]. Ethical approval was granted by a local institutional review board.

### Statistical analysis

Cohen’s effect size (w) was used to compare the socio-demographic characteristics, BMI, weight status and WC between respondents and non-respondents with a smaller Cohen’s w (<0.3) indicating a small difference [20]. Only subjects with both baseline and follow-up data were included: 2941 for BMI and 2956 for WC, which was 93.9% and 94.4% of 3131. Changes were presented by sample mean BMI and WC, and by proportions of BMI categories (18.4 or below for underweight; 18.5 to 22.9 for normal; 23 to 24.9 for overweight; 25 or above for obese) and central obesity (WC 90cm or above for men, and 80cm or above for women) [19]. The proportions of subjects in each BMI category at baseline and 3-year follow-up were examined to assess the cumulative incidence of obesity. This was contrasted with the proportion of initially overweight or obese subjects who had become normal or underweight at the 3-year follow-up. Net changes in the prevalence of general and central obesity were calculated by subtracting the number of subjects becoming normal from those becoming overweight or obese and divided by the total number of subjects. Repeated analysis was conducted among respondents age 18+ with similar findings and we presented data of all respondents in this paper.

## Results

The respondents had similar basic characteristics as non-respondents (Cohen’s w <0.3) except for a lower proportion of tertiary education attainment (Cohen’s w = 0.31) (Table 1). The mean BMI significantly increased by 0.3 percentage point from 22.8 (95% CI 22.6–22.9) at baseline to 23.1 (95% CI 23.0–23.3) at follow-up (p <0.001) (Table 2). Only few respondents (3.4% at baseline and 4.3% at follow-up) were grossly obese (BMI ≥30). Overall prevalence of overweight or obesity increased from 44.1% to 45.4% (i.e. 1.3 percentage points). The prevalence of underweight (10.0% to 8.1%, i.e, 1.9 percentage points) and overweight (20.0% to 18.9%, i.e, 1.1 percentage points) decreased but the prevalence of obesity (24.1% to 26.5%, i.e, 2.4 percentage points) and normal weight (45.8% to 46.5%, i.e, 0.7 percentage point) increased. Corresponding significant increase in mean WC (cm) by 1.5 from 79.1 (95% CI 78.8–79.5) to 80.6 (95% CI 80.2–80.9) was observed. Overall, the prevalence of central obesity increased from 28.3% to 32.4% (i.e. 4.1 percentage points) (p <0.001) with women (30.0% to 38.1%) having a non-significantly greater increase than men (23.0% to 26.1%) (p = 0.63).

**Table 1:** Comparison of baseline characteristics of respondents and non-respondents at 3-year follow-up

		Loss-follow-up (n = 3954)	Follow-up (n = 3131)	Cohen’s effect size^a^
**Age**	15–24	19.1	18.5	0.27
	25–34	17.4	10.3	
	35–44	21.6	19.8	
	45–54	17.7	23.8	
	55–64	10.4	12.7	
	65–74	8.6	10.0	
	≥75	5.1	5.0	
**Sex**	Female	51.9	53.0	0.02
	Male	48.1	47.0	
**Place of birth**	Hong Kong	63.1	54.2	0.19
	Guangdong province	23.8	31.8	
	Macau	1.0	1.3	
	Other provinces in China	9.7	10.7	
	Others	2.4	2.1	
**Marital status**	Single	33.9	28.3	0.14
	Married	58.2	64.6	
	Divorced/separated	2.7	2.1	
	Widowed	5.3	4.9	
**Education**	≤Primary	23.7	32.9	0.31
	Secondary	56.9	56.2	
	Tertiary	19.4	10.8	
**Personal income**	<$10,000	22.1	22.8	0.25
	$10,000 to $19,999	18.4	14.4	
	≥$20,000	10.5	5.8	
	No income	45.0	53.1	
	Not willing to answer	4.1	3.8	
**Central obesity**	No	79.1	71.8	0.16
	Yes	20.9	28.2	
**BMI**	Underweight (≤18.4)	11.3	10.0	0.16
	Normal (18.5–22.9)	52.4	46.0	
	Overweight (23–24.9)	17.5	19.9	
	Obese (≥25)	18.8	24.1	
**Grossly obese (BMI ≥30)**	No	97.6	96.6	0.06
	Yes	2.4	3.4	

^a^ Effects size based on Cohen’s w: 0.1 for small; 0.3 for medium; 0.5 for large

**Table 2:** Proportions of BMI and waist circumference categories at baseline and 3-year follow-up, cross-sectional analysis.

	Baseline		3-year follow-up		P values
	n	%^a^	n	%^a^	
**BMI**					
Mean BMI	2941	22.8	2941	23.1	<0.001^b^
Underweight	295	10.0	239	8.1	<0.001^c^
Normal	1348	45.8	1367	46.5	
Overweight	589	20.0	555	18.9	
Obese	709	24.1	780	26.5	
**Waist circumference**					
All (mean, cm)	2956	79.1	2956	80.6	<0.001^b^
Male (mean, cm)	1398	82.6	1398	83.8	<0.001^b^
<70	119	8.5	75	5.2	<0.001^c^
70-<80	408	29.3	424	29.6	
80-<90	549	39.3	565	39.5	
≥90	322	23.0	368	25.7	
Female (mean, cm)	1558	76.0	1558	77.7	<0.001^b^
<70	455	29.2	350	21.9	<0.001^c^
70-<80	589	37.8	641	40.0	
80-<90	367	23.6	416	26.0	
≥90	147	9.4	194	12.1	
**Central obesity**					
All	836	28.3	958	32.4	<0.001^b^
Male	322	23.0	365	26.1	<0.01^b^
Female	514	33.0	593	38.1	<0.001^b^

^a^ Column percentage unless otherwise stated.

^b^ P value from paired t-test between baseline and 3-year follow-up.

^c^ P value from signed-rank test between baseline and 3-year follow-up.

The results on individual changes of weight status showed that two-thirds of baseline normal weight (68.9%) or obese (68.7%) respondents maintained their weight status at 3-year follow-up compared with only one-third of overweight (35.3%) and less than half of underweight (44.8%) respondents (Table 3). One in 5 (22.0%) normal or underweight respondents became overweight or obese (i.e. incidence of overweight or obesity), and a similar proportion (24.8%) of overweight or obese respondents became normal or underweight. This yielded 1.3% net increase in the prevalence of overweight or obesity. Relatively more overweight respondents became obese (27.5%) than obese respondents became overweight (16.4%) (p <0.001).

Regardless of baseline categories of WC, about half the women (48.2% to 55.9%) remained in the same category at follow-up. In contrast, the proportion of men maintaining their baseline WC categories increased from 28.6% for WC <70cm to 67.4% for WC ≥90cm (p for linear trend <0.001) (Table 4). Among respondents with normal WC at baseline, 17.4% (21.1% women and 13.8% men, p <0.001) developed central obesity at follow-up. Conversely, among respondents with central obesity at baseline, 29.4% (32.6% women and 27.4% men, p = 0.11) reduced their WC to normal at follow-up. This yielded a 4.1% net increase in the prevalence of central obesity. Overall, 59.3% maintained normal WC, 12.4% developed central obesity, 8.3% resumed normal WC and 20.0% remained centrally obese (data not shown in table).

**Table 3:** Changes in weight status from baseline to 3-year follow-up (row %).

	Follow-up								
	Underweight		Normal		Overweight		Obese		P value^a^
Baseline	n	%	n	%	n	%	n	%	<0.001
Underweight	132	44.8 ^b^	132	44.8 ^c^	13	4.4 ^c^	18	6.1 ^c^	
Normal	88	6.5 ^d^	929	68.9 ^d^	218	16.2 ^c^	113	8.4 ^c^	
Overweight	11	1.9 ^d^	208	35.3 ^d^	208	35.3 ^b^	162	27.5 ^c^	
Obese	8	1.1 ^d^	98	13.8 ^d^	116	16.4 ^d^	487	68.7 ^b^	

^a^ P value from signed-rank test between baseline and 3-year follow-up.

^b^ Maintained same weight status at baseline and follow-up.

^c^ Increased BMI from baseline to follow-up.

^d^ Decreased BMI from baseline to follow-up.

**Table 4:** Changes in waist circumference from baseline to 3-year follow-up (row %).

	Follow-up (cm)								
Baseline (cm)	<70		70 to <80		80 to <90		≥90		P value^a^
Females	n	%	n	%	n	%	n	%	<0.001
<70	228	50.1 ^b^	179	39.3 ^c^	41	9.0 ^c^	7	1.5 ^c^	
70-<80	88	14.9 ^d^	329	55.9 ^b^	146	24.8 ^c^	26	4.4 ^c^	
80-<90	25	4.6 ^d^	98	26.7 ^d^	177	48.2 ^b^	75	20.4 ^c^	
≥90	6	4.1 ^d^	20	13.6 ^d^	40	27.2 ^d^	81	55.1 ^b^	
Males									<0.001
<70	34	28.6 ^b^	65	54.6 ^c^	16	13.4 ^c^	4	3.4 ^c^	
70-<80	26	6.4 ^d^	216	52.9 ^b^	136	33.3 ^c^	30	7.3 ^c^	
80-<90	10	1.8 ^d^	124	22.6 ^d^	301	54.8 ^b^	114	20.8 ^c^	
≥90	2	0.6 ^d^	10	3.1 ^d^	93	28.9 ^d^	217	67.4 ^b^	

^a^ P value was calculated from signed-rank test between baseline and 3-year follow-up.

^b^ Maintained same WC category at baseline and follow-up.

^c^ Increased WC from baseline to follow-up.

^d^ Decreased WC from baseline to follow-up.

## Discussion

Cross-sectional analysis of baseline and 3-year follow-up data found small but significant increases in mean BMI (0.3 kg^2^/m) and WC (1.5cm), prevalence of overweight or obesity (1.3 percentage points) and prevalence of central obesity (4.1 percentage points). Two repeated cross-sectional studies in adults in mainland China found a similar increase in prevalence of central obesity (4.1–4.6 percentage points) but a smaller increase in mean BMI (0.1 kg^2^/m) in 3-year intervals.[1,21] Longer-term repeated surveys (about 10 years apart) in China showed greater increases in mean BMI (0.2 to 1.2 kg^2^/m) and mean WC (1.4cm), prevalence of overweight or obesity (2.9 to 10.0 percentage points) and central obesity (2.9 to 9.2 percentage points).[22–24] However, as previously discussed, using repeated cross-sectional studies to compare changes in BMI and WC over time is problematic. Longitudinal data are preferred, but none were available in Chinese. A 10-year Japanese longitudinal study found the incidence of obesity (BMI ≥25) ranging from 5.2% to 25.6% in adults in different regions [9]. Although not directly comparable, other non-Asian studies using a higher BMI cut-off point of 30 found higher 10-year incidence rates of obesity: 15% in Australia, 12.0% in northern Sweden and 17.3% in rural areas of the United States [12,25].

More importantly, we have provided the first evidence on the short-term dynamic changes of individual weight status based on BMI and WC in the most westernised city in China. About half the respondents had changed weight status over 3 years. One-fourth had become overweight or obese, while a similar proportion had become normal. In contrast, WC changes were more pronounced for becoming normal (29.4%) than becoming centrally obese (17.4%). These results were consistent with other local findings that weight control behaviours were popular with more than one-fourth (28.8%) of respondents trying to reduce (15.5%), gain (1.3%) or maintain (12.0%) weight in the past 12 months [2]. The changes on weight status and WC may mostly happen among respondents with around-cut-off BMIs and WCs. Further studies are warranted to investigate the factors associating with the changes. Chinese studies have linked socio-demographic (sex, education) and lifestyle (smoking, alcohol, physical activities and diet) factors to weight gain [26,27]. With massive commercial advertising for weight control in Hong Kong, social pressure to be slim is great and over half the adolescents misperceived their weight status as overweight or obese [28]. On the other hand, aggressive promotion of high-energy food and lack of physical activities typical of urban living are conducive to obesity. The apparent stable prevalence of general and central obesity in Hong Kong is therefore likely due to a balance of the above opposing influences.

Our findings on short-term dynamic weight changes suggest the need to examine the factors of individual changes in weight status, and assess their impacts on morbidity and mortality in Chinese and other populations. Underweight and obesity were consistently found to be associated with diseases and mortality in other populations but the evidence was inconclusive for weight change [29–34]. Short- (3–5 years) [32,33] and long-term (12.5 years) weight changes [30] in studies using measures at 2 time-points without considering weight fluctuations in between were found to predict mortality. In contrast, long-term follow-up studies using multiple time-point assessments found non-significant associations of weight fluctuation and cycling with mortality [31,34,35]. The association with mortality is complex when weight changes occur in specific stage of the life course and when there are reverse impacts of diseases on weight change. As regards morbidity, short-term weight changes were clearly associated with increased risks of cardiovascular diseases and diabetes [36–38].

The major limitation of our study was the high attrition rate (55.8%) which may affect the representativeness of findings. However, non-response bias in the present study was unlikely to be large as respondents and non-respondents had similar baseline characteristics including weight status and central obesity. Our prevalence of weight status and central obesity was also similar to government statistics [2]. Loss to follow-up was mainly due to change in address or unsuccessful contact. Refusal was relatively uncommon (17.0%) and unlikely to be directly related to weight status as this was only one of many topics studied. Any non-response due to these common reasons probably would lead to non-differential errors [39]. Changes on weight status and waist circumference may be attributable to age, period or cohort effects. However, as data were only collected at two time point, this limits the analysis using age-period-cohort model. Finally, the analysis of general and central obesity categories were based on arbitrary cut-off values with a very small proportion being grossly obese, constraining further analysis on more extreme changes.

## Conclusions

This is one of the few studies in Chinese, which found dynamic changes (increase/stable/decrease) in weight status based on BMI and waist circumference. Future studies with better follow-up and investigating the causes of such changes of obesity indicators are warranted.
